# Sperm motility parameters as indicators of seminal quality: a comparative study between fish species

**DOI:** 10.1590/1984-3143-AR2025-0140

**Published:** 2026-07-31

**Authors:** Lorena Pacheco da Silva, Stella Indira Rocha Lobato, Rodrigo Yutaka Dishoff Kasai, Letícia Giosa Paulino, Carmen Fila Zeca Manjate, Laís Pedroso Borges, Rosicleire Veríssimo-Silveira, Alexandre Ninhaus-Silveira

**Affiliations:** 1 Laboratório de Ictiologia Neotropical – LINEO, Departamento de Biologia e Zootecnia, Faculdade de Engenharia, Universidade Estadual Paulista “Júlio de Mesquita Filho” – UNESP, Ilha Solteira, SP, Brasil

**Keywords:** aquaculture, CASA, sperm kinetic, reproductive biotechnologies, fish

## Abstract

This study investigated the patterns of sperm kinetics in two fish species, *Piaractus mesopotamicus* and *Pseudoplatystoma reticulatum* and their relationship with seminal quality. Semen samples were collected after hormonal induction, with the aim of identifying indicators of seminal quality and their relationship with reproductive success. The results revealed marked interspecific differences: *P. reticulatum* exhibited longer-lasting motility (83 ± 31 s) with stable linear movement patterns after activation, while *P. mesopotamicus* showed more intense but shorter-lasting motility (41 ± 8 s), exhibiting initial circular trajectories that transitioned to linear patterns. In *P. reticulatum*, positive correlations were found between total motility (MOT), progressivity (PROG), velocities: curvilinear (VCL), straight-line (VSL) and average (VAP), with hatching rate (HR). These are suggested to be reliable predictors of seminal quality. For *P. mesopotamicus*, multivariate analysis (PCA) indicated that sperm quality was associated with a specific combination of VCL, VAP, and amplitude and lateral displacement of the head (ALH), although no single parameter was directly correlated with HR. These differences reflect distinct evolutionary adaptations to their lotic reproductive environment, associated with effective oocyte fertilization capacity and larval hatching rate. *P. mesopotamicus* is adapted for rapid fertilization, with higher sperm speed, while *P. reticulatum* demonstrates more sustained sperm motility. These results highlight the need for species-specific approaches and demonstrate that the simple evaluation of sperm kinetic parameters in fish does not allow seminal qualification, requiring detailed knowledge of sperm movement by species, essential information to optimize the nutritional and reproductive management of breeders, and seminal cryopreservation protocols.

## Introduction

Approximately 26,000 known species of teleost fish inhabit a wide range of aquatic ecosystems (Froese and Pauly, 2019) and exhibit remarkable diversity in reproductive strategies (Helfman et al., 2009). The eco-evolutionary success of individuals depends on the interaction between reproductive traits—such as spawning behavior, larval and juvenile survival, and sexual maturation—with environmental variables, including hydrological regimes, temperature fluctuations, and resource availability (Humphries et al., 1999; Lytle and Poff, 2004). Within this context, teleosts are able to allocate energy from food intake and convert it into offspring through diverse reproductive strategies, thereby ensuring reproductive success (Vazzoler, 1996).

Most migratory fish inhabiting lotic environments form spawning aggregations during the flood season and migrate long distances upstream from feeding to spawning grounds (Godinho et al., 2010). These species are characterized by high fecundity, broadcast spawning of free-floating eggs, and the absence of parental care. Many produce demersal eggs with near-neutral buoyancy, and their embryonic development occurs rapidly as they drift downstream (Godinho et al., 2010). Thus, variability in stream conditions may alter the timing, duration, or nature of the reproductive period and behavior of migratory fish, possibly affecting trade-offs between current and future reproductive investment. However, the extent to which riverine physical conditions and behaviors during a single reproductive event influence future reproductive effort remains unclear (Larson et al., 2025).

Generally, selection is expected to favor male reproductive behaviors that enhance reproductive success. In species with external fertilization, sperm competition plays a key role (Levitan, 1998). This process, in which the sperm of two or more males compete to fertilize the same egg batch, can drive a wide array of morphological and physiological adaptations. Over evolutionary time, reproductive traits such as sperm morphology, swimming duration, and swimming speed have been shaped by natural selection (Parker and Pizzari, 2010).

Developing a detailed sperm profile requires the evaluation of semen physical properties along with sperm morphological and kinetic traits (Routray et al., 2007). Beyond fertilizing ability, sperm motility is a critical integrative indicator of quality, reflecting the efficiency of the cellular compartments responsible for motility activation and maintenance (Bobe and Labbé, 2010). However, accurately estimating sperm quality and correlating these estimators with the ability to fertilize eggs and support successful embryonic development remains challenging (Cabrita et al., 2010; Kristan et al., 2018).

Seminal quality assessment is a valuable tool for understanding the mechanisms affecting sperm function and managing factors that influence overall gamete quality (Kucharczyk et al., 2019). These factors are often linked to male reproductive performance, life history, social context, and farming conditions—including broodstock nutrition, environmental manipulation, hormone induction protocols, and semen collection and handling techniques (Cabrita et al., 2014; Nowosad et al., 2018; Gallo et al., 2022). Furthermore, sperm kinetic characterization can be used to assess the impact of reproductive biotechnologies, as such interventions may alter sperm morphology and consequently affect motility patterns (Costa et al., 2024). This enables the identification and exclusion of low-performing males from breeding programs, improving management efficiency and reducing operational costs.

The evaluation of sperm motility has been significantly enhanced by the use of Computer-Assisted Sperm Analysis (CASA) systems, which allow for objective and detailed quantification of kinetic parameters (Gallego et al., 2017; Gallego and Asturiano, 2018; Marinović et al., 2021). These systems serve as powerful tools by precisely analyzing multiple aspects of motility, including variables undetectable by manual observation (Rurangwa et al., 2004). In addition to improving reproducibility, CASA enables faster and more rigorous assessments of sperm quality. In this sense, this system has been widely used for seminal analysis of Neotropical fish species, such as the species studied in this work and described below.

*Piaractus mesopotamicus*, commonly known as pacu, is a native species of the Paraná River Basin, widely distributed across the floodplains of Brazil’s Central-West region, especially in the Pantanal wetlands (Petrere, 1989). This commercially important species is widely used in aquaculture due to its adaptive feeding behavior and tolerance to prolonged periods of food restriction (Favero et al., 2018). A rheophilic species, it undertakes upstream reproductive migrations in the wild and spawns between October and December (Baldisserotto, 2005). Eggs and larvae produced in headwaters are carried downstream to the floodplain, where they find shelter and food (Urbinati and Gonçalves, 2005). In captivity, however, reproductive success is impaired by gonadal cycle inhibition, requiring hormonal induction to stimulate spawning (Lima et al., 1991).

*Pseudoplatystoma reticulatum*, known as cachara, is one of the most commercially valuable freshwater fish species in Brazil (Crepaldi et al., 2008) and is also prized in sport fishing for its size and strength (Reid, 1983). It shows favorable traits for intensive farming and, under natural conditions, performs total spawning in upper river stretches during the rainy season, typically from November to March, when water levels, flow rates, turbulence, and oxygen concentrations are high—conditions essential for larval development (Baldisserotto, 2005; Resende et al., 1995). In captivity, however, this species does not naturally complete its reproductive cycle due to the absence of environmental stimuli associated with reproductive migration (Romagosa et al., 2004).

This study aimed to evaluate and characterize the sperm swimming behavior of *Piaractus mesopotamicus* and *Pseudoplatystoma reticulatum*, identifying potential kinetic parameters as predictors of semen quality in these species.

## Methods

Fifteen adult and sexually mature *Pseudoplatystoma reticulatum* males and twenty-two *Piaractus mesopotamicus* males were used in this study. The specimens were sourced from the Piraí fish farm, located in the municipality of Terenos, Mato Grosso do Sul, Brazil. This research was approved by the Animal Use Ethics Committee (CEUA) of the School of Engineering, UNESP, Ilha Solteira campus, under protocol no. 12/2023, in accordance with Brazilian Law 11.794/2008.

### Semen collection

Fish were hormonally induced with a single intramuscular injection of 1 mg/kg body weight of Carp Pituitary Extract (CPE), administered below the pectoral fin, following the standard protocol used by the fish farm. After 250 degree-hours (29 °C / 8.5 hours), semen was collected by gentle abdominal massage (cranial-caudal direction) using 3 mL syringes and transferred to sterile Falcon tubes. Samples contaminated with urine, feces, blood, or water were discarded. During collection, semen samples were kept chilled (4°C) in insulated boxes with ice and were analyzed immediately after the end of the collection procedure.

### Sperm characterization

#### Sperm motility

Sperm motility parameters were evaluated using a Computer-Assisted Sperm Analysis system (ISAS® Integrated Semen Analysis System, Proiser, Valencia, Spain) connected to a UB200i phase-contrast microscope (UOP/Proiser) with a 10x objective. Images were captured using an ISAS 782C camera (Proiser, Spain) and processed with the CASA software. For activation, 60 µL of 0.45% NaCl was added to 0.05 µL of semen in a Makler™ chamber (Sefi Medical Instruments Ltd., Israel). Video recording started 10 seconds post-activation, with sequential recordings taken every 5 seconds until motility dropped below 10%. CASA settings were adjusted for teleost sperm: head area between 3–20 µm^2^, frame rate of 25 frames per second, and minimum curvilinear velocity (VCL) of 10 µm/s to classify motile cells. The following motility parameters were analyzed: Total Motility (MOT, %), Progressivity (PROG, %), Average Path Velocity (VAP, μm/s), Straight-Line Velocity (VSL, μm/s), Curvilinear Velocity (VCL, μm/s), Amplitude of Lateral Head Displacement (ALH, μm), Beat Cross Frequency (BCF, Hz), Straightness (STR, %), Linearity (LIN, %), Wobble (WOB, %), and Motility Time (TM, s). All analyses were performed in triplicate to ensure accuracy.

#### Sperm concentration

For fertilization trials, sperm concentration was determined by diluting 1 µL of semen in 1000 µL of saline-formalin solution, following Ninhaus-Silveira et al. (2006). The fixed sample was homogenized, and a 10 µL aliquot was transferred to a Neubauer hemocytometer, covered with a coverslip, and left to rest for 10 minutes to allow sperm sedimentation. Sperm count was performed under a light microscope in ten fields of the chamber, and concentration was calculated following the methodology of Kavamoto and Fogli da Silveira (1986), expressed as spermatozoa per milliliter (Sptz/mL).

#### Sperm kinetics and fertilization capacity

To evaluate the influence of kinetic parameters on fertilization capacity, one female and nine males were selected. Females were hormonally induced with two doses of CPE (1 and 4 mg/kg body weight) administered 8 hours apart. Males received a single dose (1 mg/kg body weight) at the time of the second dose given to females, following the routine induction protocol of the fish farm. Gametes were collected 250 degree-hours after the second CPE dose, with part of the semen reserved for sperm analysis. For fertilization, three samples of 1600 oocytes per male were used. These were fertilized with approximately 3 µL (*P. reticulatum*) and 4 µL (*P. mesopotamicus*) of semen (±105 spermatozoa per oocyte). After dry mixing of semen and oocytes, spermatozoa were activated with 0.45% NaCl solution. Following hydration, the eggs were placed in experimental incubators (PVC; 100 mm in diameter) positioned within vertical flow-through incubators (60 L), maintained at 29°C with continuous water flow and aeration. After hatching, larvae were fixed in a solution containing 25% glutaraldehyde, 8% paraformaldehyde, and 0.2 M phosphate buffer (pH 7.2), and hatching rate (%, TE) was then calculated:


$$HR\;=\;\left(Total\;Hatched\;Larvae\;/\;Total\;Incubated\;Eggs\right)\;\times \;100$$ (1)

### Statistical analysis

Means and standard deviations were calculated to describe sperm motility parameters based on all evaluated males of *P. mesopotamicus* (*n* = 22) and *P. reticulatum* (*n* = 15). The correlation between kinetic variables and hatching rate was assessed using Pearson’s correlation test (*P* ≤ 0.05). Principal Component Analysis (PCA) was applied to reduce dimensionality, identify patterns within the dataset, and highlight variables with the greatest impact. Statistical analyses were performed using RStudio (R Development Core Team, 2022). The correlation and PCA analyses were conducted exclusively with the males used in the fertilization trials, totaling nine individuals per species.

## Results

The mean TM in*P. reticulatum*(83 ± 31 s) was approximately twice that of*P. mesopotamicus*(41 ± 8 s). The higher standard deviation in *P. reticulatum* suggests greater individual variability for this parameter, whereas *P. mesopotamicus* showed more uniform values.

**Figure 1:**
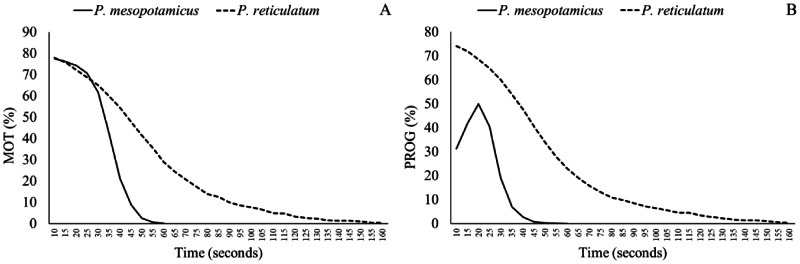
Temporal variation of MOT (Total motility) and PROG (Progressivity) in*P. mesopotamicus*and*P. reticulatum*. (A) displays the percentage of MOT over time, while (B) shows the percentage of PROG, with*P. mesopotamicus*represented by solid lines and*P. reticulatum*by dashed lines.

At 10 seconds post-activation, MOT (Figure 1A) was similar between*P. mesopotamicus*(77 ± 23%) and*P. reticulatum*(78 ± 21%). However, within the first 25 seconds, the decline in this parameter occurred more slowly in *P. mesopotamicus* compared to *P. reticulatum*. After this period, motility in *P. mesopotamicus* dropped sharply, reaching less than 10% by 45 seconds, while in *P. reticulatum*, the decrease occurred more gradually, maintaining 48 ± 23% motile spermatozoa at the same time point, highlighting differences in motility duration between species.

PROG was lower in *P. mesopotamicus* (Figure 1B), with an initial average of 31 ± 15%, representing less than half of the average observed in *P. reticulatum* (74 ± 21%). Between 10 and 20 seconds, *P. mesopotamicus* showed an increase in the number of progressively moving cells, peaking at 50 ± 21%, followed by a subsequent decrease. In contrast, *P. reticulatum* exhibited a continuous decrease in this parameter throughout the activation period.

**Figure 2:**
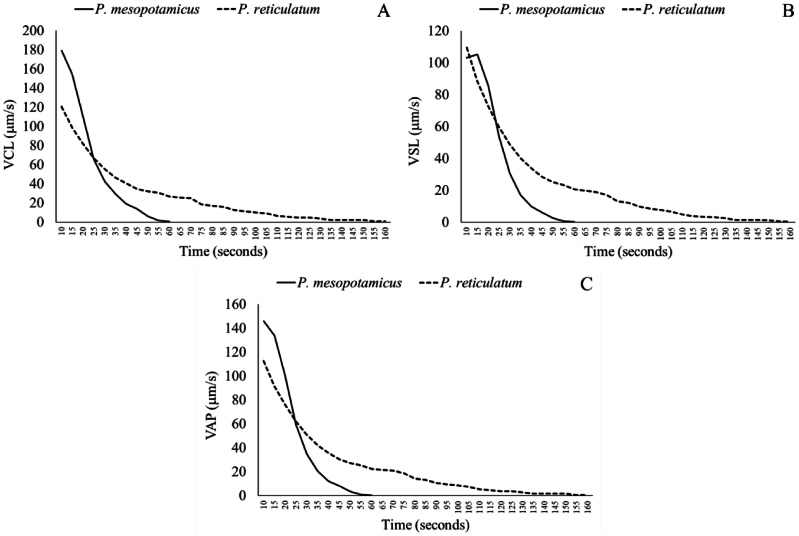
Temporal dynamics of sperm motility parameters (VCL, VSL, and VAP) in*P. mesopotamicus*and*P. reticulatum*. (A) shows curvilinear velocity (VCL, μm/s), (B) displays straight-line velocity (VSL, μm/s), and (C) presents average path velocity (VAP, μm/s) over time, with solid lines representing*P. mesopotamicus*and dashed lines indicating*P. reticulatum*. The y-axes show velocity scales (μm/s) while x-axes represent time (seconds).

Regarding sperm velocity parameters, as shown in Figure 2, VCL in *P. mesopotamicus* was higher, with a mean of 179 ± 40 μm/s at 10 seconds post-activation, followed by a sharp decrease in the subsequent seconds, dropping below 10 μm/s at 50 seconds, with a mean of 6 ± 12 μm/s. In *P. reticulatum*, the initial VCL was 121 ± 29 μm/s, with a steeper decline up to 35 seconds (47 ± 11 μm/s), followed by a more gradual reduction thereafter.

VSL in *P. mesopotamicus* showed a mean of 103 ± 23 μm/s at 10 seconds, slightly lower than that of *P. reticulatum* (110 ± 25 μm/s). However, between 10 and 15 seconds, *P. mesopotamicus* exhibited a 2 μm/s increase, followed by a rapid decrease. In *P. reticulatum*, there was a sharp decline between 10 and approximately 40 seconds (34 ± 11 μm/s), after which the values stabilized.

VAP followed a similar pattern to VCL, although with smoother transitions. In *P. mesopotamicus*, the initial average was 146 ± 28 μm/s, with a steep decline starting at 15 seconds. In *P. reticulatum*, the initial average was 113 ± 25 μm/s at 10 seconds, decreasing more rapidly until 35 seconds, then more gradually afterward.

**Figure 3:**
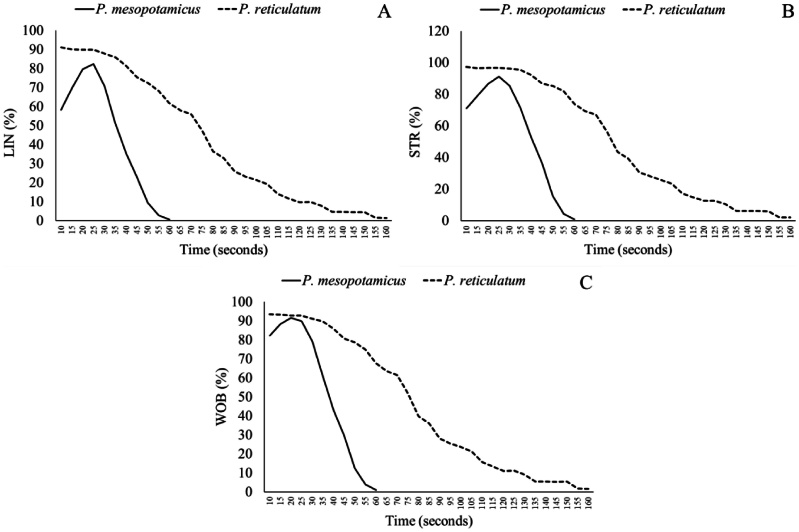
Sperm motility kinematics (LIN, STR, and WOB) in*P. mesopotamicus*and*P. reticulatum*over time. (A) illustrates linearity (LIN, %), (B) shows straightness (STR, %), (C) displays wobble (WOB, %). Solid lines represent*P. mesopotamicus*, while dashed lines denote*P. reticulatum*. The y-axes indicate the respective motility parameters (%), and the x-axes represent time (seconds).

LIN, shown in Figure 3A, displayed distinct behaviors between species. In *P. mesopotamicus*, the initial mean was 58 ± 10%, increasing between 10 and 25 seconds, reaching 82 ± 11%. In *P. reticulatum*, the highest value was observed at 10 seconds (91 ± 5%) and decreased gradually throughout the activation period, remaining higher than that of *P. mesopotamicus* during the entire evaluation.

STR (Figure 3B) followed a pattern similar to LIN. In *P. mesopotamicus*, the initial mean was 71 ± 9%, increasing up to 25 seconds and peaking at 91 ± 6%, followed by a decline. In *P. reticulatum*, the initial mean was significantly higher at 97 ± 3% and remained relatively constant during the first time points, with slight increases below 1% at some points, and began to decrease only after 40 seconds.

WOB also showed an initial increase in *P. mesopotamicus* (Figure 3C), from 82 ± 11% at 10 seconds to 92 ± 6% at 20 seconds, followed by a steep decline after 25 seconds. In *P. reticulatum*, the initial mean at 10 seconds was 94 ± 3%, and like LIN and STR, the decline occurred gradually throughout the observation period.

**Figure 4:**
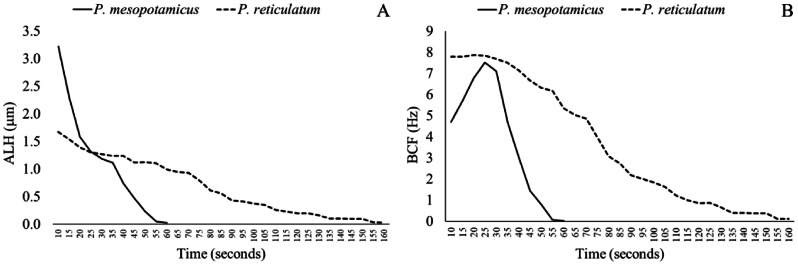
Temporal dynamics of sperm motility parameters (ALH and BCF) in*P. mesopotamicus*and*P. reticulatum*. (A) shows amplitude of lateral head displacement (ALH, µm), and (B) displays beat-cross frequency (BCF, Hz) over time. Solid lines represent*P. mesopotamicus*, while dashed lines indicate*P. reticulatum*. The y-axes depict ALH (µm) and BCF (Hz), respectively, and the x-axes show time progression (seconds).

ALH showed a similar decreasing pattern throughout the motility period for both species, as shown in Figure 4A. However, differences in mean values were observed, with the mean at 10 seconds being 3 ± 2 µm in *P. mesopotamicus* and 2 ± 0.4 µm in *P. reticulatum*.

BCF exhibited a pattern similar to the other previously evaluated parameters (Figure 4B). In *P. mesopotamicus*, the mean BCF at 10 seconds was 5 ± 1 Hz, increasing to 8 ± 1 Hz at 25 seconds, followed by a progressive decrease. In *P. reticulatum*, the mean values were higher, with 8 ± 1 Hz at 10 seconds, showing an initial stabilization phase until approximately 30 seconds (8 ± 0.5 Hz), followed by a gradual decline until the end of sperm motility.

**Figure 5:**
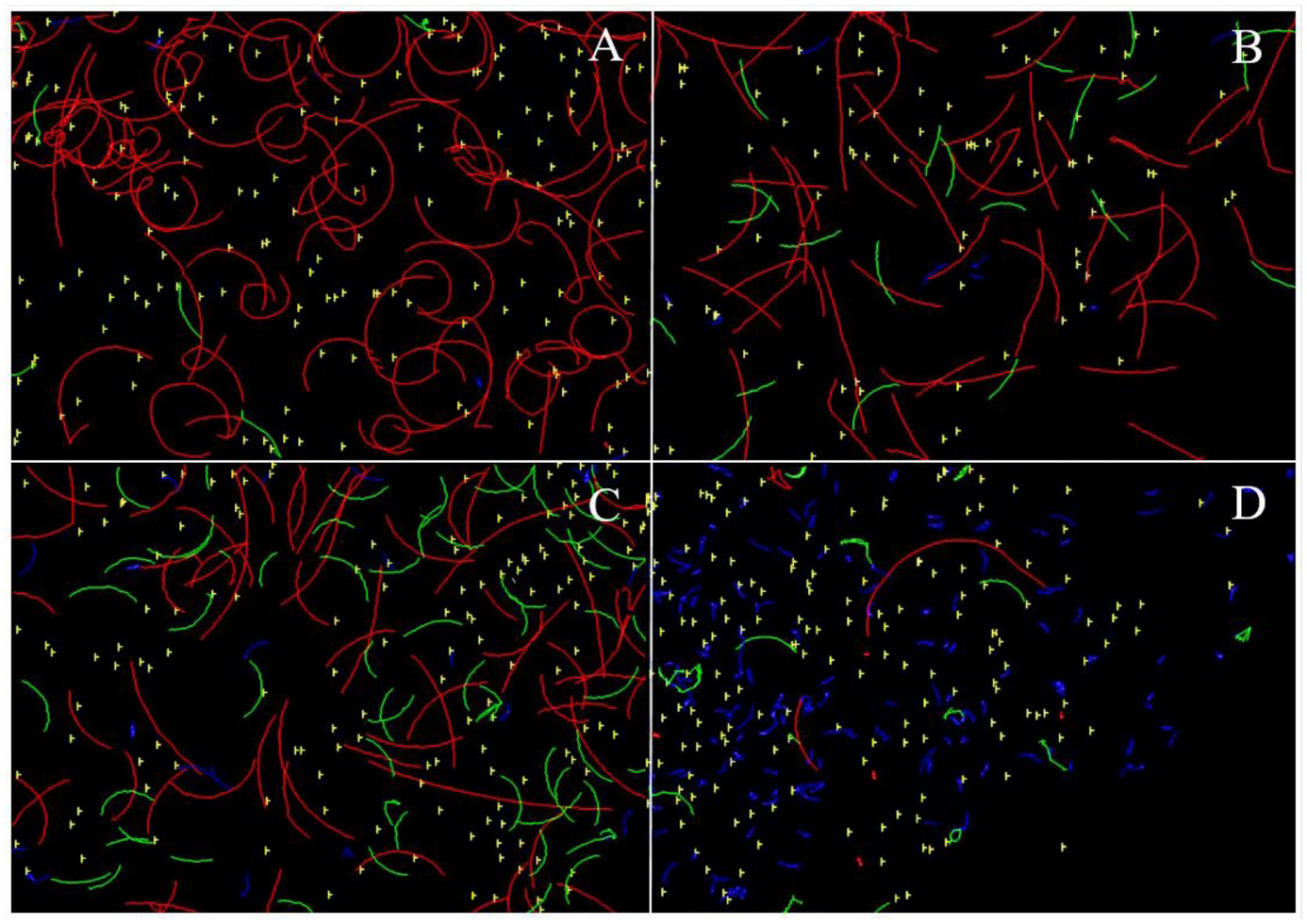
Computer-Assisted Analysis of Sperm Trajectories and Velocities in *P. mesopotamicus* Over Time. Computer-Assisted Sperm Analysis (CASA) of *P. mesopotamicus* spermatozoa activated with 0.45% NaCl, showing progressive changes in swimming trajectories and velocity distributions over time. (A) 10 sec post-activation, (B) 20 sec, (C) 30 sec, and (D) 40 sec. Trajectories are color-coded by velocity: red (fast; >80 µm/s), green (medium; 40-80 µm/s), and blue (slow; 10-40 µm/s).

The combination of these parameters results in a distinctive visual motility pattern for each of the analyzed species. In *P. mesopotamicus*, at 10 seconds (Figure 5A), spermatozoa exhibited predominantly circular and irregular movement, with only a few showing linear trajectories. Most were moving rapidly, as indicated by the red lines. At 20 seconds (Figure 5B), this pattern became more linear for a large portion of the population, accompanying the increase in several kinetic parameters, although the velocity remained high. At 30 seconds (Figure 5C), the trajectories remained mostly linear or slightly curved, but with a higher proportion of medium and slow velocities. By 40 seconds (Figure 5D), a marked decrease in motility was observed, with most spermatozoa displaying slow movement with no defined pattern.

**Figure 6:**
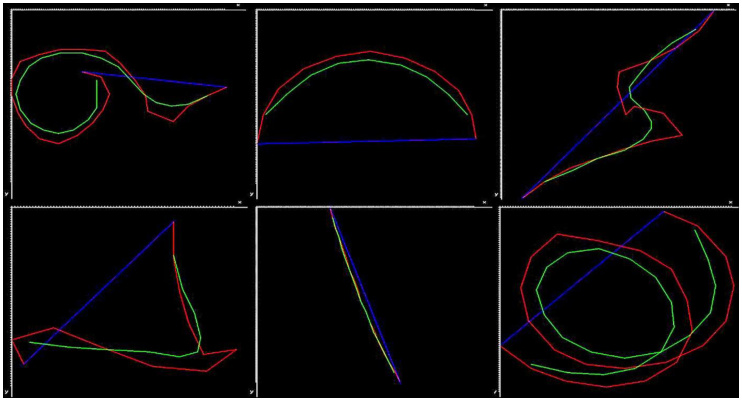
Kinematic Analysis of Individual *P. mesopotamicus* Spermatozoa 10 Seconds Post-Activation. Computer-Assisted Sperm Analysis (CASA) of individual *P. mesopotamicus* spermatozoa 10 seconds after activation, showing representative velocity vectors. Red lines depict curvilinear velocity (VCL, µm/s); blue lines indicate straight-line velocity (VSL, µm/s); and green lines display average path velocity (VAP, µm/s).

For *P. mesopotamicus*, the individual analysis revealed a variety of sperm trajectory patterns, as illustrated in Figure 6. Observed paths included undefined shapes, as well as circular, semicircular, “S” - shaped, and linear trajectories.

**Figure 7:**
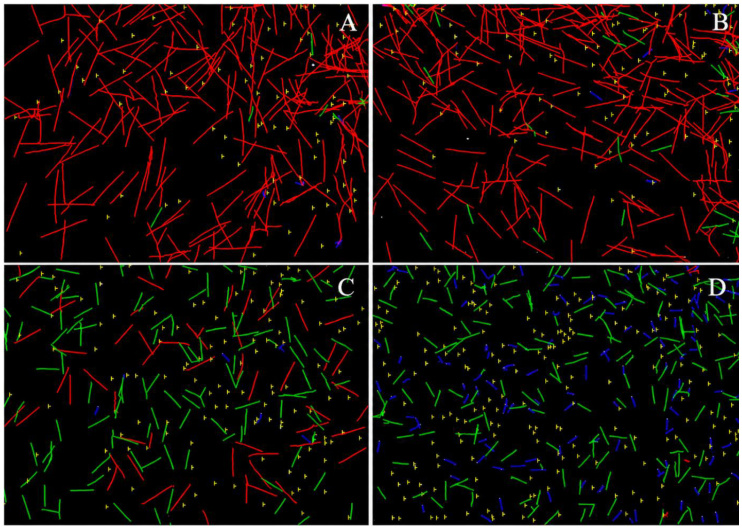
Computer-Assisted Analysis of Sperm Trajectories and Velocities in *P. reticulatum* Over Time. Computer-Assisted Sperm Analysis (CASA) of *P. reticulatum* spermatozoa activated with 0.45% NaCl, showing progressive changes in swimming trajectories and velocity distributions over time. (A) 10 sec post-activation, (B) 20 sec, (C) 30 sec, and (D) 40 sec. Trajectories are color-coded by velocity: red (fast; >50 µm/s), green (medium; 25-50 µm/s), and blue (slow; 10-25 µm/s).

*P. reticulatum* exhibited a well-defined and linear movement pattern from the beginning of activation (Figure 7), with nearly the entire population showing fast displacement. At 20 seconds (Figure 7B), this linear pattern persisted, with a predominance of fast movements and few spermatozoa at medium speeds. At 30 (Figure 7C) and 40 seconds (Figure 7D), the trajectories remained linear, but velocity began to vary, with a predominance of medium and slow movements. However, unlike *P. mesopotamicus*, a significant portion still maintained considerable speed at 40 seconds.

**Figure 8:**
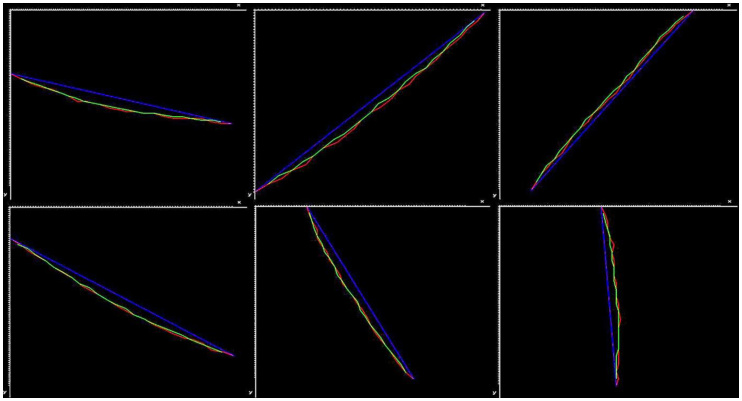
Kinematic Analysis of Individual *P. reticulatum* Spermatozoa 10 Seconds Post-Activation. Computer-Assisted Sperm Analysis (CASA) of individual *P. reticulatum* spermatozoa 10 seconds after activation, showing representative velocity vectors. Red lines depict curvilinear velocity (VCL, µm/s); blue lines indicate straight-line velocity (VSL, µm/s); and green lines display average path velocity (VAP, µm/s).

The individual sperm analysis of *P. reticulatum* highlighted the uniformity in movement, showing a well-established linear pattern across the entire sperm population (Figure 8).

The average sperm concentration obtained in *P. mesopotamicus* was 35.45 ± 8.8 x 10^9^ spermatozoa/mL, while in *P. reticulatum* it was 43.98 ± 7.8 x 10^9^ spermatozoa/mL. The hatching rate (HR) in *P. mesopotamicus* was 82.26 ± 7.91%, slightly lower than the 98.60 ± 1.16% observed in *P. reticulatum*.

Pearson’s correlation analysis for *P. mesopotamicus* (Table 1) did not reveal positive correlations between HR and the evaluated kinetic parameters. However, significant correlations were identified among the kinetic parameters themselves, suggesting the influence of certain variables on the motility pattern in this species. Specifically, LIN showed a positive correlation with BCF, which was also positively associated with PROG. Additionally, WOB showed a negative correlation with ALH, while ALH exerted a positive influence on all analyzed velocity parameters.

**Table 1:** Pearson correlation between hatching rate and the kinetic variables of *P. mesopotamicus* and *P reticulatum* spermatozoa used in oocyte fertilization.

	**HR**	**TM**	**MOT**	**PROG**	**VCL**	**VSL**	**VAP**	**LIN**	**STR**	**WOB**	**ALH**
**P. mesopotamicus**											
**TM**	-0.01										
**MOT**	0.08	-0.13									
**PROG**	-0.02	0.48*	0.03								
**VCL**	0.14	0.17	0.18	0.16							
**VSL**	0.09	0.39*	0.04	0.70*	0.80*						
**VAP**	0.19	0.11	0.11	0.22	0.97*	0.83*					
**LIN**	-0.03	0.41*	-0.17	0.94*	0.00	0.61*	0.11				
**STR**	-0.08	0.54*	-0.08	0.96*	0.10	0.66*	0.14	0.96*			
**WOB**	0.18	-0.25	-0.35	0.23	-0.30	0.02	-0.07	0.44*	0.16		
**ALH**	-0.03	0.28	0.23	-0.09	0.85*	0.51*	0.73*	-0.29	-0.09	-0.66*	
**BCF**	-0.26	0.33	0.00	0.57*	-0.30	0.09	-0.36	0.54*	0.64*	-0.17	-0.23
**P. retculatum**											
**TM**	0.23										
**MOT**	0.55*	0.73*									
**PROG**	0.55*	0.66*	0.96*								
**VCL**	0.47*	0.68*	0.86*	0.80*							
**VSL**	0.45*	0.59*	0.80*	0.85*	0.92*						
**VAP**	0.47*	0.65*	0.85*	0.84*	0.98*	0.97*					
**LIN**	-0.14	-0.33	-0.28	-0.02	-0.36	0.04	-0.19				
**STR**	-0.12	-0.27	-0.23	0.05	-0.26	0.12	-0.11	0.96*			
**WOB**	-0.16	-0.37	-0.32	-0.08	-0.43*	-0.06	-0.26	0.95*	0.83*		
**ALH**	0.31	0.54*	0.62*	0.47*	0.75*	0.50*	0.63*	-0.72*	-0.55*	-0.85*	
**BCF**	-0.20	-0.26	-0.30	-0.22	-0.22	-0.15	-0.22	0.21	0.26	0.11	-0.15

Legends: HR: Hatching rate; TM: Motility time; MOT: Total motility; PROG: Progressivity; VCL: Curvilinear velocity; VSL: Straight-line velocity; VAP: Average path velocity; LIN: Linearity; STR: Straightness; WOB: Wobble; ALH: Amplitude of lateral head displacement; BCF: Beat cross frequency. The values presented correspond to correlation coefficients, with significance considered at *P* ≤ 0.05. Positive correlations indicate direct associations between variables, while negative correlations indicate inverse associations. Significant values are highlighted in bold and marked with an asterisk (*).

In *P. reticulatum*, HR showed a positive correlation with MOT, PROG, VCL, VSL, and VAP, as shown in Table 1. Similarly to *P. mesopotamicus*, ALH had a positive effect on all sperm velocities, as well as on MOT, PROG, and TM. However, ALH showed a negative correlation with LIN, STR, and WOB. In this species, BCF did not show significant correlations with the other evaluated parameters.

**Figure 9:**
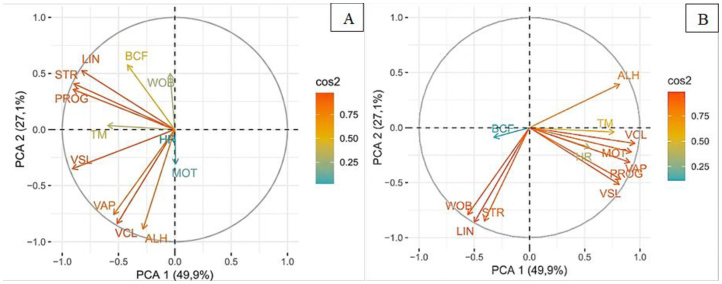
Principal Component Analysis of the 12 parameters evaluated for *P. mesopotamicus* (A) and *P. reticulatum* (B)*.* Hatching rate (HR). Motility time (TM). Total motility (MOT). Progressivity (PROG). Curvilinear velocity (VCL). Straight-line velocity (VSL). Average path velocity (VAP). Linearity (LIN). Straightness (STR). Wobble (WOB). Amplitude of lateral head displacement (ALH). and Beat cross frequency (BCF). The color scale bar represents the intensity of the correlation of each parameter with the first and second principal componentes.

Since PCA considers the parameters as a whole. it allows the observation of patterns and correlations different from those identified individually by the Pearson correlation. In *P. mesopotamicus* (Figure 9). for example. although HR and MOT had a low contribution to the analysis (indicated by the blue coloration). they were positively correlated with each other and negatively correlated with WOB. Additionally. HR was also correlated with ALH. VCL. and VAP — relationships not previously detected in the individual correlation analyses.

In *P. reticulatum*. HR showed a direct correlation with VAP. visualized by the overlapping vectors. It also showed correlations with VCL. MOT. VAP. PROG. and VSL. thus reinforcing the data obtained in the previous analysis (Figure 9).

## Discussion

Despite progress in this field. detailed descriptions of sperm and flagellar movement patterns in fish remain scarce and often contradictory. which hinders the interpretation of the effects of certain biotechnologies on sperm kinetics. In externally fertilizing species such as teleosts. spermatozoa are released directly into the water and must reach and enter the oocyte micropyle to achieve fertilization (Browne et al., 2015). In other words. the first stage of natural selection occurs even before gamete encounter. and the selective pressure associated with external fertilization is undeniably higher (Kholodnyy et al., 2020).

The diversity of spawning environments. ranging from sheltered areas to turbulent waters. imposes different risks of gamete dispersion (Balon, 1975). This factor is particularly relevant for the species evaluated in this study. both adapted to environments with strong currents. where gametes need to act quickly during fertilization. as they can be easily carried away by the water.

Notably. *P. reticulatum* and *P. mesopotamicus* exhibited distinct sperm swimming strategies. The former showed more sustained motility (higher TM) and a more gradual decline in MOT. which may favor fertilization under conditions of extended exposure to the oocyte. In contrast. *P. mesopotamicus* showed similar initial motility. with a sharp reduction after 25 seconds. suggesting a fast fertilization strategy. Values reported by Maria et al. (2004) and Miliorini et al. (2002) for *P. mesopotamicus* corroborate this trend. although they show wide variation in motility duration. The values for *P. reticulatum* are consistent with those obtained by Sanchez (2023). This information is crucial for optimizing sperm activation time in in vitro fertilization procedures. Although the rapid decline in motility parameters after the onset of flagellar movement is a conserved pattern in fish due to energy depletion (Cosson. 2019). the species evaluated here manage this energy reserve differently.

Although total motility (MOT) remains the most widely used parameter for sperm evaluation (Rurangwa et al., 2004). its predictive capacity may vary between species. In the present study. both species showed high hatching rates (>80%); however. only in *P. reticulatum* was a direct association between MOT and HR observed. reinforcing the idea that MOT alone may be a reliable indicator of semen quality in this species. In *P. mesopotamicus*. no such direct correlation was found.

*P. mesopotamicus* exhibited a high initial energetic investment. characterized by higher VCL values. which may represent an advantage in highly turbulent environments. However. the absence of a direct correlation between VCL and hatching rate (HR) in this species indicates that velocity is secondary to the need for synchronized activation and close physical proximity to the oocyte at the moment of spawning. as occurs in in vitro fertilization. where parameters such as ALH and MOT may play more determinant roles.

In contrast. the kinetics of *P. reticulatum* revealed a more conservative and sustained profile (higher TM). The significant correlation between all velocity parameters (VCL. VSL. VAP) and larval hatching rate indicates that maintaining progressivity over time is a strong determinant of semen quality in this species. This finding corroborates data from other Prochilodontidae. such as *Prochilodus lineatus* (Viveiros et al.. 2010). supporting the use of velocity parameters as reliable predictors of reproductive success in this species. Native species such as *Brycon hilarii* (Costa, 2022) may exhibit intermediate behaviors. characterized by slightly sinuous. not very fast but vigorous trajectories. with chaotic movement and frequent changes in swimming pattern. intermediate between those observed in *P. reticulatum* and *P. mesopotamicus*.

The kinetic differences observed between the species may reflect distinct adaptive strategies in the face of sperm competition — common in externally fertilizing species. where sperm dilution and environmental variation are high (Liao et al., 2018). In such scenarios. traits such as higher swimming speed and progressive motility tend to be favored (Snook, 2005; Parker et al., 2010). Moreover. studies show that sperm behavior can be plastically adjusted in response to competition cues (Zajitschek et al., 2014; Rudolfsen et al., 2006; Bartlett et al., 2017). reinforcing the possibility of different responses even among species sharing similar habitats.

CASA parameter analysis revealed that ALH and BCF are critical modulators of directionality. In *P. reticulatum*. the balance between propulsion force and stability resulted in more directed swimming (higher LIN and STR). In contrast. *P. mesopotamicus* exhibited a more circular and irregular initial pattern. transitioning to more linear trajectories with a transient increase in BCF. This plastic adjustment. similar to that observed in *Colossoma macropomum* (Gallego et al.. 2017). may suggest that initial circular swimming is a strategy to keep spermatozoa closer to the oocyte for a longer period. facilitating micropyle entry.

The progressive movement of spermatozoa depends on the ability of the flagellum to generate waves that originate at the junction between the head and tail and propagate along its length. enabling efficient displacement (Dzyuba et al.. 2017). In this context. PROG. LIN. and STR were consistently higher in *P. reticulatum*. indicating a more directed and stable swimming pattern throughout sperm activity. This swimming profile has been associated with higher fertilization rates in other species such as *Sparus aurata* (Beirão et al., 2011) and *Brycon cephalus* (Bashiyo-Silva. 2014). On the other hand. *P. mesopotamicus* showed lower initial values for these parameters. with increases over time. This adjustment was accompanied by a transient increase in BCF. LIN. and STR. Although linearity and progressivity are widely related to semen quality in mammals (Ferraz et al., 2014; Muiño et al., 2008). in fish these associations are more variable. For example. in *Colossoma macropomum*. fast. non-progressive sperm with more circular trajectories showed greater correlation with fertilization (Gallego et al., 2017). demonstrating behavior similar to that observed for *P. mesopotamicus* in this study. where. despite lower average values. good hatching rates were observed.

The behavior observed in *P. mesopotamicus* follows the opposite trend to that found in *Merlucius merlucius* (Cosson et al., 2010). where sperm began their activity with more linear trajectories and later transitioned to increasingly narrow circular swimming patterns over time. The increase in circular sperm movement tends to reduce the volume of water explored. limiting their dispersion and. consequently. the probability of encountering the oocyte. However. this more restricted swimming pattern may increase the chance of hitting the micropyle. favoring fertilization in specific situations (Cosson et al., 2010). This change in swimming pattern may be influenced by factors such as the collection method and temperature of the activating medium. as observed in *Salmo salar* (Merino et al., 2024). These effects. which vary between species. may enhance or impair reproductive performance. Additional factors such as oocyte morphology. sperm concentration. and dispersion dynamics around the micropyle also affect fertilization (Suquet et al., 1995; Rurangwa et al., 1998).

In addition to interspecific variation in sperm behavior. the literature indicates that kinetic profiles may also vary intrinsically according to the social status of the breeder. In *Oncorhynchus tshawytscha*. juvenile and dominant males exhibit trajectories with different degrees of linearity. a divergence shaped by sperm competition and distinct fertilization tactics (Rosengrave et al.. 2024). This may help explain the standard deviation observed in the present study. particularly for *P. reticulatum*.

## Conclusion

The study demonstrates that *P. reticulatum* and *P. mesopotamicus* exhibit distinct sperm swimming strategies. despite both reproducing in lotic environments. The former species is characterized by linear and steady movement. whereas the latter shows a more circular and oscillatory pattern. While semen quality in *P. reticulatum* is predicted by total motility. progressivity. and velocity parameters (VCL. VSL. and VAP). in *P. mesopotamicus* it appears to be associated with a more complex combination of ALH and velocities. as elucidated by PCA analysis. Understanding these species-specific patterns is essential for improving broodstock nutritional and reproductive management. refining cryopreservation protocols. and optimizing larval hatching rates in aquaculture.
